# Laryngeal Radiation Fibrosis: A Case of Failed Awake Flexible Fibreoptic Intubation

**DOI:** 10.1155/2011/878910

**Published:** 2011-12-19

**Authors:** Johannes M. Huitink, Lambert Zijp

**Affiliations:** ^1^Department of Anaesthesiology, VU University Medical Center, 1081 HV Amsterdam, The Netherlands; ^2^Department of Radiotherapy, Antoni van Leeuwenhoek Hospital-The Netherlands Cancer Institute, 1066 CX Amsterdam, The Netherlands

## Abstract

Awake fibreoptic intubation is accepted as the gold standard for intubation of patients with an anticipated difficult airway. Radiation fibrosis may cause difficulties during the intubation procedure. We present an unusual severe case of radiation induced changes to the larynx, with limited clinical symptoms, that caused failure of the fibreoptic intubation technique. A review of the known literature on radiation fibrosis and airway management is presented.

## 1. Introduction

Awake fibreoptic intubation is sometimes very challenging in patients with head and neck cancer. We would like to present an interesting case of radiation-induced changes to the upper airway and review the known literature.

## 2. Case Presentation

A 48-year-old male patient was scheduled for a total laryngectomy with bilateral neck dissection because of recurrent laryngeal cancer. He was treated for laryngeal cancer three years earlier with radiation therapy. On preoperative evaluation, it was noted that the patient was edentate and had a trismus with a limited mouth opening of only two centimetres. There was severe induration of the neck, which was caused by prior radiotherapy to this region. He had no stridor, and his breathing pattern was unobstructed.

Because of an anticipated difficult airway, an awake flexible fibreoptic intubation was scheduled.

At the beginning of the awake intubation procedure, patient was given a continuous propofol 1% infusion of 15 mL/hr for sedation after topical analgesia of the nasopharynx and larynx. The airway was uneventful anaesthetized with lignocaine spray 2% and 10%. The introduction of the bronchoscope (Olympus LF-TP) through the nose was easy, and uneventful and vision was clear. The epiglottis was swollen and deformed. Passing of the vocal cords with the insertion cord was problematic, and it was noted that the airway seemed very narrow at glottic level. The airway was not reactive because of sufficient analgesia and prior radiotherapy. Because of patient discomfort, we tried to railroad the tracheal tube (Mallinckrodt MLT 6.0) swiftly over the bronchoscope. However, a resistance was encountered. At the same moment the patient had a gagging reflex, and the upper airway became acutely obstructed. Percutaneous measured oxygen saturation rapidly decreased to 60%, and it was decided to perform an emergency coniotomy with a Quicktrach 1.0 (Rusch Germany, 4.0 mm, uncuffed) device that was available on the difficult intubation trolley. The insertion of the device was fast, but ventilation of the patient was difficult and the oxygen saturation decreased further to 40%. There was no time to assemble the Manujet (VBM Company) device to oxygenate the patient.

With a scalpel, an incision was made by the head and neck surgeon just below the coniotomy site, and a cuffed tube 5.0 was inserted over a gum elastic boogie with difficulty into the trachea. After intubation of the trachea, the condition of the patient stabilized quickly, and it was decided to proceed with the total laryngectomy and neck dissection as planned.

**Figure 1:**
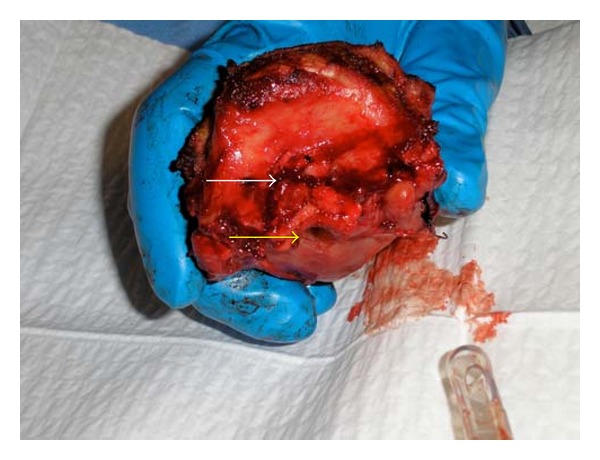
Larynx with tumour mass after dissection. White arrow: tumour mass. Yellow arrow: barely recognizable epiglottis and severely narrowed glottic opening. Tracheal tube size 6.0.

After dissection of the larynx and on inspection of the anatomical specimen, it was noted that the airway lumen was severely narrowed. In theatre, it was tried to pass the same tracheal tube 6.0 through the glottis, which was impossible, because of extensive fibrosis and severe narrowing in airway diameter (Figure 1).

After the procedure the patient was transferred to the intensive care unit, and recovery was uneventful without neurological, pulmonal, or cardiovascular complications.

**Figure 2:**
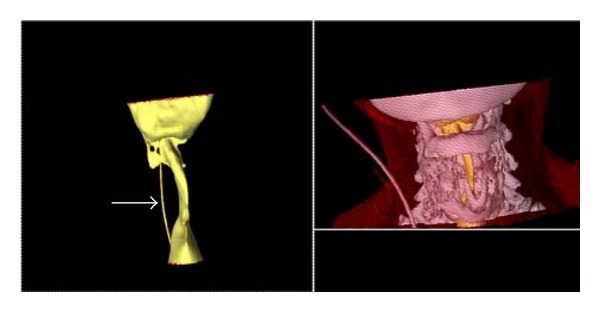
Three dimensional CT reconstruction of the larynx, yellow structures are air. Gray areas are bony structures. There is narrowing of the airway of 4 mm over a 4 cm long trajectory at the subglottic level. White arrow: reconstruction of air column in the feeding tube which is present in the oesophagus.

The next day a three-dimensional upper airway reconstruction of the preoperative CT scan was performed. It was noted that the larynx was severely narrowed over a four-centimetre-long trajectory. The smallest airway diameter measured was four millimetres (mm) (Figure 2).

After anatomical and histological examination of the specimen by the pathologist, it was reported that the smallest airway diameter was four mm, and the trachea showed signs of oedema and fibrosis. There were signs of recurrent laryngeal carcinoma and osteoradionecrosis of the tracheal rings and thyroid bone.

## 3. Discussion

In this case report, a patient with recurrent laryngeal cancer and a difficult airway had to undergo emergency tracheotomy because of a failed awake flexible fibreoptic intubation. This patient was treated three years earlier with radiotherapy. Awake fibreoptic intubation is the gold standard for many patients with a difficult airway; however, it has its limitations. Reasons for failure of fibreoptic intubation are many among them loss of vision due to bleeding or mucous, tumour size, or severe upper airway narrowing that makes insertion of the cord of the bronchoscope impossible [1–5].

In most patients, we prefer to use a flexible bronchoscope of sufficient size because intubation is easier than with smaller scopes [6]. The insertion cord diameter of the Olympus LF TP is only 5.1 mm. Therefore, we normally use a tube 6.0 because that fits very tight around the insertion cord and permits adequate ventilation during often prolonged procedures. The majority of adult patients in our practise can be safely intubated with this tube size.

During the intubation procedure, we could not pass the fibrosis with the insertion cord of the flexible scope nor tracheal tube, which is an unusual situation. This problem, however, caused acute laryngospasm and airway obstruction that mandated emergency coniotomy.

Patients with head and neck malignancies are commonly treated by a combination of surgery and adjuvant chemotherapy and/or radiotherapy. The degree of airway changes due to the radiation varies from patient to patient. Radiotherapy induces oedema with subsequent fibrosis or necrosis in the exposed tissues. These changes may affect the buccal mucosa, bone dentition, and larynx [7–9]. Bag mask ventilation may be difficult due to osteoradionecrosis of the mandible or lack of dentition. Direct laryngoscopy in these patients is rendered difficult by fibrosis, oedema, and restriction of mouth opening by trismus. The epiglottis may be completely deformed or oedematous, which makes visualization of the glottis difficult. Osteoradionecrosis is a severe complication of a high dose and frequent radiation to vascular bone. The mandible, being highly vascular, is more susceptible than other bones. Osteoradionecrosis occurs secondary to impairment of revascularisation in bone tissue and the severity of the damage depends on total dose of radiation, frequency of exposure, and prior trauma [10].

Very severe airway narrowing is infrequently encountered in head and neck cancer patients. The incidence is not known but we estimate that we see 10–15 patients per year (>600 head and neck cancer procedures per year) with very severely narrowed upper airways.

During bronchoscopy, it may be difficult to visually estimate the severity of an airway obstruction, because of augmentation through the lenses of the bronchoscope. Structures appear much bigger than they really are. This holds especially true for situations in which a suboptimal view is obtained due to mucous or blood. In contrast, during direct laryngoscopy or videolaryngoscopy, it is easier to estimate if the tip of the tube can pass the obstruction because both targets, the glottis and the tip of the tube, appear in the same field of vision. During fibreoptic intubation however, it is not possible to visualize the outside of the tube during intubation, because the tube is railroaded over the insertion cord. Sometimes it may be easier to use a guiding catheter through the suction canal of the bronchoscope. This way it can be seen whether a small catheter can be passed through a small opening and also if the remaining space around the catheter will be sufficient to accommodate a larger tracheal tube [11, 12].

When airway narrowing is too severe to pass a tracheal tube, a tracheotomy after local analgesia is the technique of choice to secure the airway. However, most head and neck surgeons do not prefer to do this prior to a total laryngectomy.

Three-dimensional CT reconstructions could have been performed before the procedure; however, this is not a standard procedure, and, in this patient, we only had the CT scan that was available from another hospital. It was not yet available in our computer system. There are not many medical centres in the world where this is routinely performed. Only, when a patient presents with an inspiratory stridor, which is a warning sign for severe upper airway narrowing, this is sometimes done when a recent CT scan is available.

Our patient had severe upper airway narrowing. Had a tracheal tube been chosen that was smaller, we would have had to use a paediatric size tube 4.0 that is not of sufficient length, or an Aintree intubation catheter, which can only be used for a short-time period for oxygenation. If we had chosen a smaller size bronchoscope, it would have been possible to pass the vocal cords, but advancement of the tracheal tube would have given the same difficulties. Normally, in most patients with airway obstruction, it is possible to manipulate the oedema or soft tissue tumours with some slight force or manipulation of the tube; however, after radiotherapy, upper airways may become severely obstructed and hard as wood, which was the case in our patient. Rigid scope intubation was not an option because of the fixed limited mouth opening.

It is a rather difficult clinical decision to estimate if a patient with a difficult airway but no other clinical signs of airway obstruction needs to undergo a tracheotomy under local analgesia.

There are no studies that provide guidelines for decision making on performance of a tracheotomy under local anaesthesia, only anecdotic case reports. Some patients present to the emergency room or theatre without warning signs other than dyspnoea or difficulty breathing. Not all patients have an inspiratory stridor. Our patient had none of these symptoms.

Videolaryngoscopy may be of help to give an indication of the severity of the airway problems if mouth opening is sufficient which is not the case in our patient was. A videolaryngoscopic-assisted fibreoptic intubation would probably have warned us for the impeding airway disaster [13].

In their review of the management of head and neck cancer patients, a series of more then 800 patients in 10-year time at a single institution, Moorthy et al. describe a preintubation fibreoptic evaluation of the larynx [14]. They use a grading score of the tumour and manage the patients according to the evaluation. This elegant technique, however, may be difficult if most of the tumour or airway narrowing is located on a level below the glottis.

Three-dimensional virtual airway reconstruction and navigation is a valuable tool when available. When airway size is very small, an appropriate tube is necessary. Another problem is that very small tubes do not fit around adult intubating bronchoscopes or are too small to ventilate the patient for a prolonged period of time.

High-frequency jet ventilation is dangerous in patients with severely obstructed airways because of the risk of barotrauma. In some patients with laryngeal tumours, a tracheotomy can be prevented by (laser) debulking when the tumorous lesions are soft. However, the trachea needs to be intubated most of the times during these procedures.

For airway rescue in this patient, we could not follow the existing difficult airway guidelines, a supraglottic airway device would not have solved the ventilation problem.

The lesson we can learn from this case is that extreme caution is necessary when the upper airway is severely fibrosized because of prior radiotherapy. An indurated neck region is a good predictive sign for a difficult direct laryngoscopy, because the base of the tongue has not enough space within the submandibular area. A soft tracheal tube has not enough rigidity to pass these airways. If our patient would have had enough mouth opening, we probably would have used a videolaryngoscope and a small stiff intubating catheter to pass the obstructed airway.

In summary in patients with severe upper airway narrowing because of radiation fibrosis, an awake fibreoptic intubation may be impossible and a tracheotomy is the only means of securing this airway; however, there may be no evident warning signs that the airway lumen is too narrow. Diagnostic imaging with CT scanning or MRI may detect these patients with severely narrowed airways without clinical symptoms.
